# Susceptibility of High-Manganese Steel to High-Temperature Cracking

**DOI:** 10.3390/ma15228198

**Published:** 2022-11-18

**Authors:** Gabriela Fojt-Dymara, Marek Opiela, Wojciech Borek

**Affiliations:** 1 Department of Engineering Processes Automation and Integrated Manufacturing Systems, Silesian University of Technology, 18A Konarskiego Street, 44-100 Gliwice, Poland; 2 Department of Engineering Materials and Biomaterials, Silesian University of Technology, 18A Konarskiego Street, 44-100 Gliwice, Poland

**Keywords:** high-manganese steel, hot ductility, cracking, nil strength temperature, nil ductility temperature, ductility recovery temperature

## Abstract

Tests were carried out on two high-Mn steels: 27Mn-4Si-2Al-Nb with Nb microaddition and 24Mn-3Si-1.5Al-Nb-Ti with Nb and Ti microadditions. High-manganese austenitic steels, due to their good strength and plastic properties belong to the AHSS (Advanced High-Strength Steel) group and are used in the automotive industry. The main difficulties faced during the casting of the steel and hot working are hot cracks, which can appear in the surface of the ingot. Cracks on the edges of the sheet after hot rolling are the reason for cutting the edges of the sheet and increasing production costs and material losses. The main reason for the formation of hot cracks is the decrease in metal ductility in the high-temperature brittleness range (HTBR). The width of the HTBR depends on mechanical properties and microstructural factors, i.e., non-metallic inclusions or intermetallic phases at austenite grain boundaries. In this paper, a hot tensile test was performed. The research was performed on the GLEEBLE 3800 thermomechanical simulator. This test allows us to determine the width of the high-temperature brittleness range (HTBR), the Nil Strength Temperature (NST), the Nil Ductility Temperature (NDT), and the Ductility Recovery Temperature (DRT). Hot ductility was determined from the value of the reduction in area R(A). The obtained results make it possible to determine the temperature of the beginning of hot working from the tested high-Mn steels. Fractographic research enabled us to define mechanisms of hot cracking. It was found that hot cracks form as a result of disruptions in the liquid film on crystals’ boundaries.

## 1. Introduction

High-manganese austenitic steels are very modern materials used in the automotive industry and belong to the group of advanced high-strength steels. They are characterized by a very good combination of strength and plastic properties and a reduced density of 7.3 g/cm^3^. They contain between 15% and 30% Mn and additions of Si and Al, as well as micro-additions of Nb, Ti, V, and B [1,2].

Thanks to their properties, they are used in the production of automotive parts that absorb a large amount of energy during a collision, ensuring the safety of the driver and passengers, for example, a motor longitudinal member or a crossbeam. Thanks to their reduced density, fuel consumption and the emission of harmful exhaust gases can be reduced [3,4]. The precursor of high-manganese steels was Robert Abbott Hadfield, who, in 1882, patented a steel grade containing 1.2% C and 12% Mn, characterized by high abrasion resistance as a result of hardening during plastic deformation.

Contemporary high-manganese steels are divided into three groups: TRIP steels (Transformation-Induced Plasticity), TWIP steels (Twinning-Induced Plasticity), and TRIPLEX steels. This division is related to the content of elements, the microstructure, the strengthening mechanism, and the stacking fault energy (SFE) value [5,6]. Depending on the content of Mn, Al, and Si, these steels show a specific SFE value and a characteristic deformation mechanism, such as deformation-induced martensitic transformation (TRIP effect) [7,8], deformation-induced mechanical twinning (TWIP effect) [9,10], and deformation-induced formation of micro-shear bands (MBIP effect) [11,12,13,14]. Despite a very good set of mechanical properties, these steels have not been used for industrial production so far. This is due to the complications associated with the processes of casting and hot forming. In the continuous casting of steel, during straightening operations, the subsurface area of the ingot is subject to complex thermoplastic stresses. The surface of the ingot is subjected to intensive water cooling and the cyclical action of guide rollers. If the steel does not exhibit high hot ductility, there is a high probability of crack initiation and propagation on the surface of the ingot; hence, the use of these steels as a construction material on an industrial scale is associated with an increase in high-temperature ductility. In order to determine the susceptibility of steel to high-temperature cracking, the following values are determined: the zero-strength temperature, the plasticity recovery temperature, and the high-temperature brittleness range (HTBR), i.e., the range between the zero-strength temperature and the plasticity recovery temperature. This range is determined on the basis of tests of mechanical properties. The wider the range, the more susceptible the steel is to hot cracking [15,16,17,18,19,20].

The phenomenon of high-temperature brittleness can be observed analogously in welding processes. A similar effect occurs in the weld pool when the metal solidifies. Prokhorov [17,18,19] showed in his research that the upper limit of the high-temperature brittleness range coincides with the liquidus temperature, and the lower one is slightly below the solidus temperature. Moreover, he determined that the high-temperature cracking was influenced by the strain growth rate (stress), the plastic deformation ability of steel, and the size of the high-temperature brittleness range.

Determining the high-temperature brittleness range will allow us to avoid high-temperature cracking in austenitic high-manganese steels for the automotive industry. Starting hot working below the recovery temperature will increase the production efficiency of products from these steels.

Hence, the aim of this work is to determine the high-temperature plasticity characteristics of steels (NST, NDT, DRT) and to assess the effect of the micro-addition of Ti on the high-temperature brittleness range (HTBR) in order to develop conditions for the thermo-plastic treatment of sheets with high mechanical properties and guaranteed hot ductility. The results of scanning microscopy studies of two high-manganese steels with different concentrations of Mn, Si, and Al and micro-additions of Ti and Nb allowed us to determine the fracture surface of the samples.

## 2. Materials and Methods

The tests were carried out on two high-manganese steels, 27Mn-4Si-2Al-Nb and 24Mn-4Si-1.5Al-Nb-Ti, the chemical compositions of which are presented in Table 1. Additionally, a micro-addition of Ti was introduced into the 24Mn-4Si-1.5Al-Nb-Ti steel. The plastic deformation process using Gleeble 3800 thermomechanical simulator (Dynamic Systems Inc. Poestenkill, NY, USA) was carried out on specimens with a diameter of 6 mm and a length of 81 mm.

In order to assess the susceptibility of the tested steels to cracking at high temperature, the following were determined:

- Nil strength temperature (NST);
- Nil ductility temperature (NDT);
- Ductility recovery temperature (DRT).

**Figure 1:**
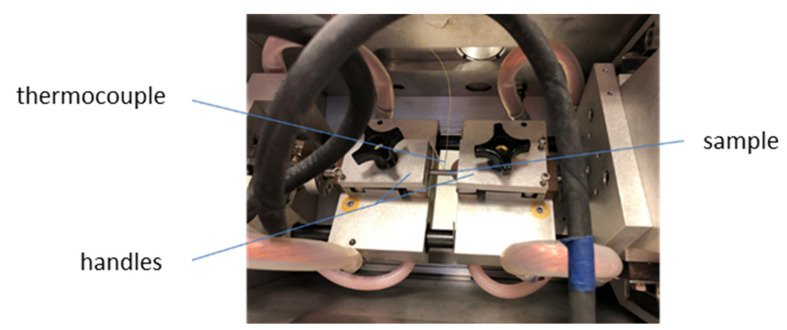
View of the measuring system for determining the NST (Nil Strength Temperature).

Tests in the Gleeble 3800 thermomechanical simulator made it possible to evaluate the tendency of the tested steels to high-temperature cracking. The temperature of nil strength (NST), the temperature of nil ductility (NDT), and the ductility recovery temperature (DRT) were determined. Moreover, the high-temperature brittleness range (HTBR) was determined, which, according to reports in the literature, is the interval between the temperature of nil strength and the temperature of ductility recovery [20,21,22]. The wider the high-temperature brittleness range, the greater the tendency of the material to form cracks. First, the temperature of nil strength was determined, which is defined as the temperature at which the steel completely loses its strength during heating [16,22,23,24,25]. At the temperature of nil strength and above, the material is not able to bear any loads [16]. The temperature of nil strength, determined experimentally, corresponded to the highest value of the temperature recorded during the test when the material lost its stability, which was accompanied by a clear decrease in the measured temperature. The temperature of the loss of strength (NST) was determined using a special measuring system, which is shown in Figure 1. In order to determine the NST, the sample was heated to a temperature of 1200 °C at a rate of 20 °C/s; after this temperature was exceeded, the heat was increased at a rate of 1 °C/s, and a slight tensile force (approx. 80 N) was applied until it cracked. The NST value is the temperature at which the sample cracked.

**Figure 2:**
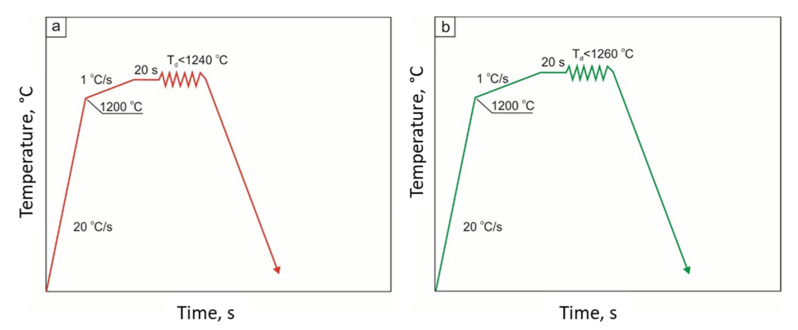
Scheme of determining the nil ductility temperature (NDT) in 27Mn-4Si-2Al-Nb steel (**a**) and in 24Mn-3Si-1.5Al-Nb-Ti steel (**b**).

**Figure 3:**
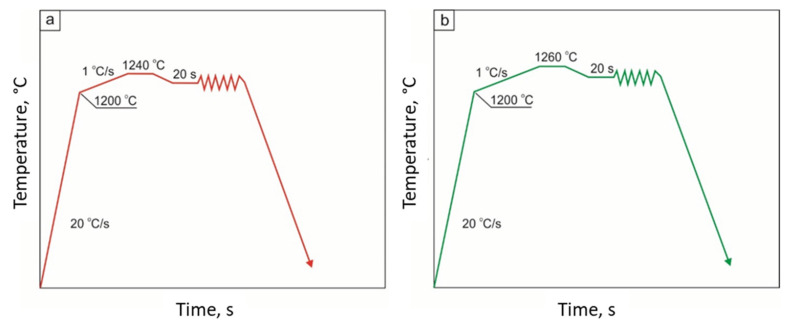
Scheme of determining the ductility recovery temperature (DRT) in 27Mn-4Si-2Al-Nb steel (**a**) and in 24Mn-3Si-1.5Al-Nb-Ti steel (**b**).

The nil ductility temperature (NDT) and the ductility recovery temperature (DRT) were determined according to the diagrams presented in Figure 2 and Figure 3, respectively. In order to determine the NDT temperature, the samples were heated to a temperature of 1200 °C at a rate of 20 °C/s, and then the tensile temperature rate was set to 1 °C/s. The highest deformation temperature was 1240 °C for 27Mn-4Si-2Al-Nb steel (Figure 2a) and 1260 °C for 24Mn-3Si-1.5Al-Nb-Ti steel (Figure 2b).

Then, the ductility recovery temperature (DRT) was determined according to the scheme shown in Figure 3. The samples were heated to a temperature of 1200 °C at a rate of 20 °C/s, and then to a temperature of 1240 °C (27Mn-4Si-2Al-Nb steel) and 1260 °C (24Mn-3Si-1.5Al-Nb-Ti steel) at a rate of 1 °C/s. After holding at this temperature, which should be 10 °C lower than the NST, the specimens were cooled to the target temperature and deformed. The highest applied tensile temperature was 1230 °C for 27Mn-4Si-2Al-Nb steel and 1250 °C for 24Mn-3Si-1.5Al-Nb-Ti steel.

The fracture surfaces of the samples after cracking were carried out with a ZEISS high-resolution SUPRA 35 scanning electron microscope (Carl Zeiss AG, Oberkochen, Germany) using an accelerating voltage of 20 kV and a magnification ranging from 100× to 15,000×.

## 3. Results

**Figure 4:**
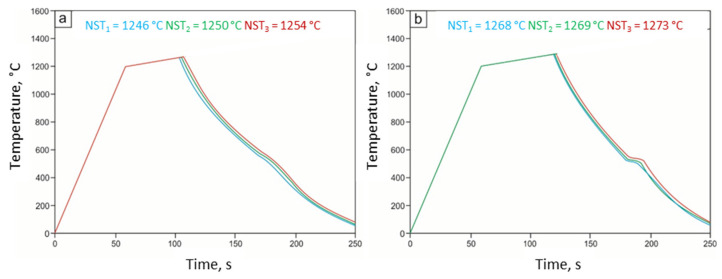
Diagrams of temperature changes as a function of time allowing for the determination of NST for steel: 27Mn-4Si-2Al-Nb (**a**) and 24Mn-3Si-1.5Al-Nb-Ti (**b**).

The results of the experiment for the analyzed steels are shown in Figure 4 in the form of diagrams of temperature changes as a function of time.

**Figure 5:**
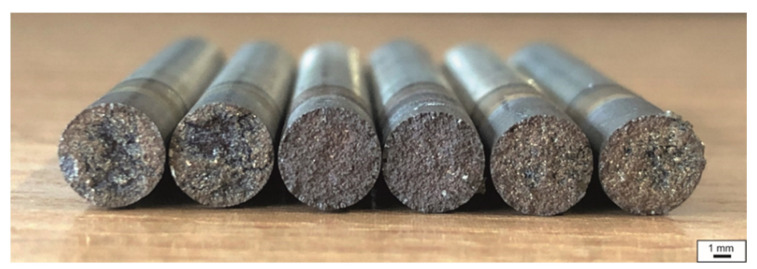
Fracture surfaces of samples of 27Mn-4Si-2Al-Nb steel after the experiment to determine the NST.

The average value of the NST was determined from three measurements. The average NST value for 27Mn-4Si-2Al-Nb steel is 1250 °C, while the average NST value for 24Mn-3Si-1.5Al-Nb-Ti steel is 20 °C higher and amounts to 1270 °C. For example, Figure 5 shows the view of samples made of 27Mn-4Si-2Al-Nb steel after the experiment.

**Figure 6:**
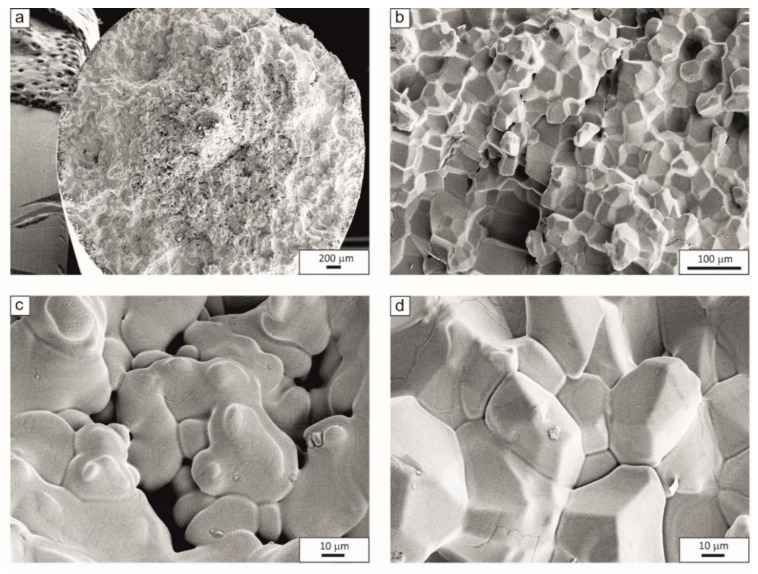
Fracture surfaces of samples of 27Mn-4Si-2Al-Nb steel after cracking at NST_2_ = 1250 °C. (**a**) brittle fracture, (**b**) intercrystalline fracture area, (**c**,**d**) melted grain boundaries.

**Figure 7:**
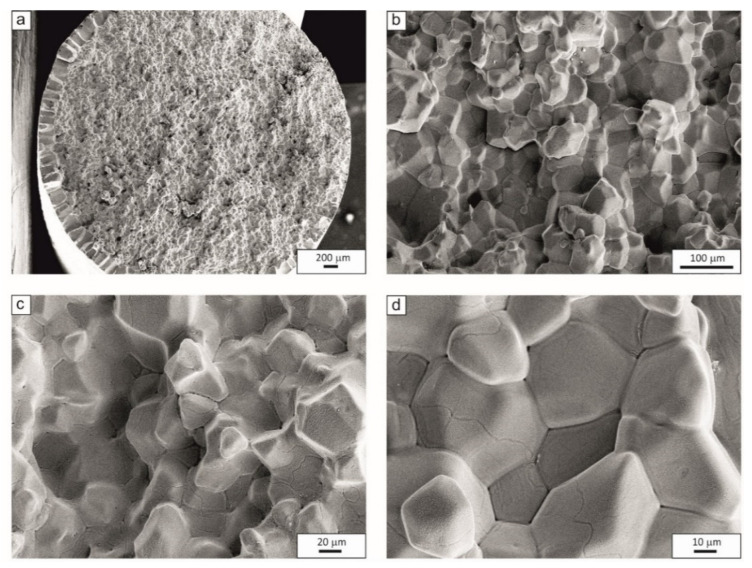
Fracture surfaces of samples of 24Mn-3Si-1.5Al-Nb-Ti steel after cracking at NST_2_ = 1269 °C. (**a**) brittle fracture, (**b**) intercrystalline fracture area, (**c**,**d**) melted grain boundaries.

The fracture surfaces after cracking at a temperature NDT_2_ = 1250 °C for 27Mn-4Si-2Al-Nb steel and NDT_2_ = 1269 °C for 24Mn-3Si-1.5Al-Nb-Ti steel are shown in Figure 6 and Figure 7, respectively. Scanning electron microscopy studies in the area of loss of cohesion show local melting of austenite grain boundaries. On the surface of the fractures, brittle fractures and transcrystalline and intercrystalline cracks were found (Figure 6d and Figure 7d).

Next, the nil ductility temperature (NDT) and the ductility recovery temperature (DRT) of the tested steels were determined. The experiment enabling the determination of the NDT was carried out in accordance with the diagram shown in Figure 2a for the 27Mn-4Si-2Al-Nb steel and, for the 24Mn-3Si-1.5Al-Nb-Ti steel, according to the diagram shown in Figure 2b. These tests consisted of heating the samples to a temperature of 1200 °C at the rate of 20 °C/s and then to the desired tensile temperature at the rate of 1 °C/s. According to the literature [17,18,19,20,21,22,23,24,25,26], the steel loses its plastic deformation capacity at the moment of reaching the NDT. It is assumed that steel loses its plasticity when the reduction in area R(A) value is ≤1% [20].

On the other hand, the ductility recovery temperature (DRT) was determined according to the assumptions presented in Figure 3a,b—for 27Mn-4Si-2Al-Nb and 24Mn-3Si-1.5Al-Nb-Ti steels, respectively. These tests consisted of heating the samples to the temperature of 1200 °C at the rate of 20 °C/s, then heating to a temperature 10 °C lower than the NST at the rate of 1 °C/s, cooling to the target temperature, and applying tensile force. It is assumed that the DRT is the temperature at which the reduction in area R(A) value is ≤5% [16,17,21].

**Figure 8:**
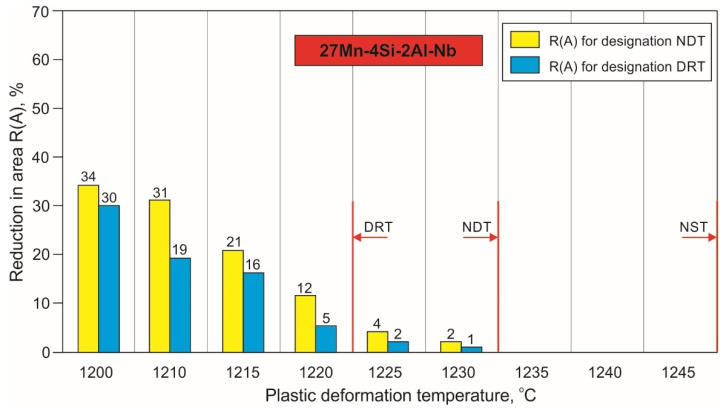
Influence of plastic deformation temperature on the reduction in area R(A) of samples of 27Mn-4Si-2Al-Nb steel after tensile force according to the scheme shown in Figure 2a and Figure 3a.

**Figure 9:**
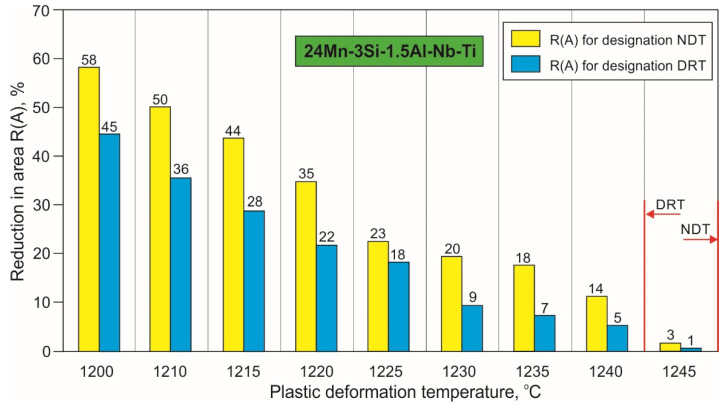
Influence of plastic deformation temperature on the reduction in area R(A) of samples of 24Mn-3Si-1.5Al-Nb-Ti steel after tensile force according to the scheme shown in Figure 2b and Figure 3b.

Based on the reduction in area R(A) analysis, the NDT and DRT were determined. The influence of the plastic deformation temperature on the narrowing of the tested steel samples, tensile according to the assumptions (Figure 2 and Figure 3), is shown in Figure 8 and Figure 9. As it results from the data presented in Figure 8, 27Mn-4Si-2Al-Nb steel loses its plasticity in a relatively narrow temperature range. The R(A) values presented in this figure, being the average of three tests, indicate that the temperature at which the 27Mn-4Si-2Al-Nb steel did not show plasticity is 1235 °C. On the other hand, a narrowing of 5% was obtained by a sample of this steel deformed at a temperature of 1220 °C (DRT). Furthermore, gradually lowering the temperature results in an increase of the R(A) to approx. 34% at the temperature of 1200 °C.

**Figure 10:**
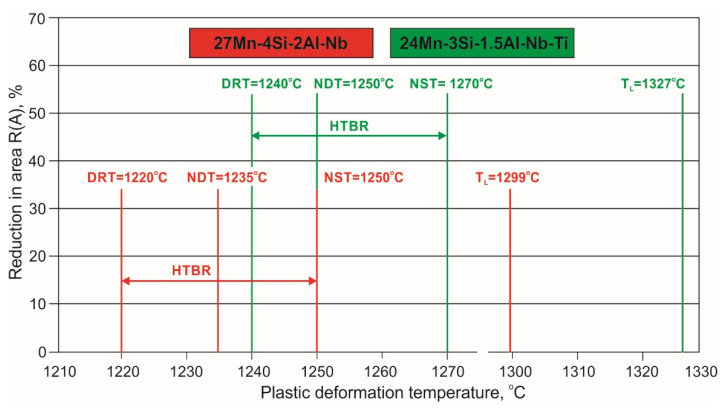
High-temperature plasticity characteristics and the high-temperature brittleness range (HTBR) of the tested steels.

The results of the tests enabling the determination of the NDT and DRT for the 24Mn-3Si-1.5Al-Nb-Ti steel are shown in Figure 9. In contrast to the 27Mn-4Si-2Al-Nb steel, the steel with the micro-addition of Ti shows an easy reduction in area gradient in the temperature range used and significantly higher R(A) values at the same temperatures. The 24Mn-3Si-1.5Al-Nb-Ti steel also shows higher NDT and DRT values of 1250 °C and 1240 °C, respectively. The contraction at the lowest applied temperature (1200 °C) is almost twice as high as that of 27Mn-4Si-2Al-Nb steel. A collective summary of the high-temperature plasticity characteristics of the tested steels with the designated high-temperature brittleness ranges (HTBR) is shown in Figure 10.

The temperature of loss of plasticity during the heating of 27Mn-4Si-2Al-Nb steel is NDT = 1235 °C, while this steel regains its plasticity during cooling at DRT = 1220 °C. The determined indices for the 24Mn-3Si-1.5Al-Nb-Ti steel are NDT = 1250 °C and DRT = 1240 °C. The designated high-temperature brittleness range of 27Mn-4Si-2Al-Nb steel is 30 °C and is within the temperature range of 1220–1250 °C. Although the HTBR of 24Mn-3Si-1.5Al-Nb-Ti steel is also 30 °C, it is moved to a higher temperature and is in the range of 1240–1270 °C.

**Figure 11:**
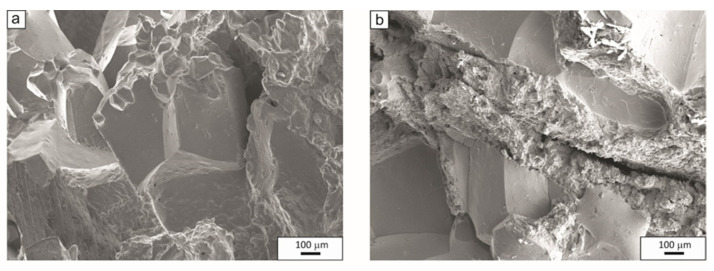
Fracture surfaces of samples of 27Mn-4Si-2Al-Nb steel after tensile test at temperatures NDT = 1235 °C (**a**) and DRT = 1220 °C (**b**).

**Figure 12:**
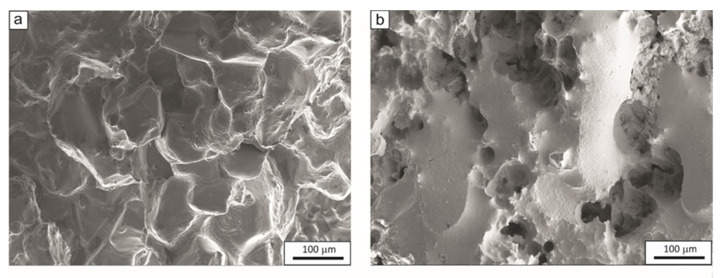
Fracture surfaces of samples of 24Mn-3Si-1.5Al-Nb-Ti steel after tensile test at temperatures NDT = 1250 °C (**a**) and DRT = 1240 °C (**b**).

The tensile fractures of samples at NDT and DRT temperatures are shown in Figure 11 and Figure 12, respectively. Figure 11a shows the tensile fracture surface of the sample made of 27Mn-4Si-2Al-Nb steel at 1235 °C = NDT with locally melted austenite grains, flat surfaces, and a local fracture along the austenite grain boundary. On the other hand, Figure 11b shows the surface of a sample of this steel after tensioning at a temperature of 1220 °C = DRT. The surface of the sample shows the characteristics of an intercrystalline fracture with a locally occurring ductile fracture. The sample has an R(A) value of 5% and demonstrates recovery of plasticity. Figure 12 shows the fracture surface of the samples made of 24Mn-3Si-1.5Al-Nb-Ti steel after deformation at the temperatures of 1250 °C = NDT and 1240 °C = DRT. After applying tensile force to this steel at the nil ductility temperature, the melting of austenite grains and a few cracks were observed on the surface (Figure 12a). On the other hand, the surface of the sample after tensile force was applied at the temperature of ductility recovery shows the features of a mixed ductile–brittle fracture, with visible flat areas, voids, and cavities (Figure 12b).

## 4. Discussion

The high-temperature tensile tests carried out in the Gleeble 3800 thermomechanical simulator also allowed us to determine the nil strength temperature (NST), the nil ductility temperature (NDT), the ductility recovery temperature (DRT), and the high-temperature brittleness range (HTBR) of the tested steels. The determined average value of NST for the 27Mn-4Si-2Al-Nb steel is 1250 °C, and for the 24Mn-3Si-1.5Al-Nb-Ti steel, it is 1270 °C. The obtained values are close to the average NST values for high-manganese steels [16,21,25,26,27,28]. The tests carried out allowing the determination of the NST and DRT have shown that the 24Mn-3Si-1.5Al-Nb-Ti steel is characterized by significantly higher contraction values in the entire temperature range (Figure 9) compared to the 27Mn-4Si-2Al-Nb steel (Figure 8). The values of the NDT and DRT for 27Mn-4Si-2Al-Nb steel are 1235 °C and 1220 °C, respectively. The values of these temperatures for the 24Mn-3Si-1.5Al-Nb-Ti steel are higher and amount to 1250 °C and 1240 °C, respectively (Figure 10). The determined high-temperature brittleness range for both steels is 30 °C, while the 24Mn-3Si-1.5Al-Nb-Ti steel’s HTBR is at a higher temperature (Figure 10).

Similar tests for the evaluation of high-temperature ductility were carried out by Kuc et al. in [26]. They observed the microstructure of high-manganese steel containing 30% Mn and 9% Al. During the tests on the Gleebe 3800 simulator, NST = 1255 °C and DRT = 1215 °C were determined. Based on the analysis, the values of the narrowing were determined by NDT 1235 °C, thus determining the tendency of the steel to cracking during the tensile test. During the thermoplastic processing, the austenite recrystallized and the ferrite healed. At a temperature of 1225 °C, they revealed interdendritic cracks, which could indicate the appearance of a liquid phase at the grain boundaries. They observed a cleavage crack in the area of the dendrites. Below the temperature of 1225 °C, the cracks were brittle, intercrystalline, and transcrystalline. As a result of further cooling, they observed an intercrystalline fracture and an increase in plasticity. The highest plastic properties were achieved by high-manganese steel with Al addition in the temperature range from 800 °C to 1150 °C, showing ductile cracking. The obtained test results made it possible to develop a continuous casting process and to select the most favorable thermoplastic processing parameters for the tested high-manganese steel.

27Mn-4Si-2Al-Nb steels has more additional Al than 24Mn-3Si1.5Al-Nb-Ti, and it is more susceptible to hot cracking, the same results were given Adamiec et al. [27]. Steel containing 24% Mn and 5.5% Al is more prone to hot cracking than steel containing 17% Mn and 3% Al. The authors conducted a study of the susceptibility of high-manganese steel with an austenitic structure to high-temperature cracking and observed that high-temperature cracks in steel containing 17% Mn and 3% Al appeared at a deformation above 5%, while in steel containing 24% Mn and 5.5% Al, cracks occurred at a deformation above 0.77%. They showed that steel containing 17% Mn and 3% Al is resistant to high-temperature cracking, and steel containing 24% Mn and 5.5% Al is prone to hot cracking. In the initial stage of crystallization, plasticity was high due to the participation of liquid and solid phases and thus the free movement of the liquid. As it solidified, the plasticity decreased to a minimum value. The reason for this was the build-up of the solid phase and the limitation of the movement of the liquid, which formed low-melting eutectics at the crystal boundaries. In the final stage of crystallization, the strength and plasticity increased again. However, below the solidus temperature, the strength of steel decreased as a result of the formation of crystal defects, changes taking place, and the separation of dispersion phases at the grain boundaries. Cracks could also be caused by the formation of a new mesh of grain boundaries that passed through the original crystals. The authors concluded that hot cracks are formed as a result of the action of shear stresses causing sliding along the new grain boundaries and the grouping of dislocations and vacancies.

Yang et al. [29] obtained similar results. The high-temperature tensile tests were carried out on a Gleeble-3500 thermal simulator in the temperature range of 600–1310 °C. The high-manganese Fe-Mn-C-Al TWIP/TRIP steel at different temperatures was studied. In the whole tested temperature range, the reductions in area have values from 47.3% to 89.4%. The nil high-temperature ductility ranged from 1275 °C to the melting point temperature, and the range of medium-temperature ductility was 1000–1250 °C. Other research [30,31] points out that the crack sensitivity will greatly increase when the R(A) value is less than 40%, and steel cracks do not easily appear when the R(A) value is greater than 60% [32].

Recently, there have been few studies on the susceptibility of high-manganese steels to high-temperature cracking, which will affect the surface cracking of ingots during the hot rolling and continuous casting process. Lan et al. [33] carried out the hot rolling on Fe-22Mn-0.7C TWIP steel in the temperature range of 700–1250 °C and found that the reductions in area were extremely low, all below 40%. Franceschi et al. [34] carried out a heat treatment for a new steel with the TRIP effect. The steel showed intercritical annealing at 780 °C and tempering at 400 °C for 30 min. Higher hardness and higher tensile strength were obtained using isothermal quenching. The martensite in the microstructure was responsible for the low ductility [35]. Mou et al. [36] found that lamellar austenite was more likely to cause stress relaxation during martensitic transformation, resulting in low plasticity.

## 5. Conclusions

The high requirements of the automotive industry for steel products in car construction leads to the development of research works aimed at shaping their strength and plastic properties. First of all, these properties relate to ensuring the hot ductility of the steel at the initial temperature of the reduction in the continuous ingot’s cross-section and during straightening operations.

The conducted experiments on the Gleeble 3800 thermomechanical simulator allowed us to determine the fracture tendency of the tested high-manganese steels 27Mn-4Si-2Al-Nb and 24Mn-3Si-1.5Al-Nb-Ti during tensile tests.

Based on the research, the following conclusions can be drawn:

- The tested high-manganese steels have a relatively narrow high-temperature brittleness range, amounting to 30 °C;
- It was found that the tested steels are characterized by relatively low values of the determined NST, NDT, and DRT compared to low-alloy steels;
- The HTBR, which is the difference between the zero-strength temperature and the ductility recovery temperature for 27Mn-4Si-2Al-Nb steel is in the range of 1250 °C to 1220 °C;
- The HTBR for 24Mn-3Si-1.5Al-Nb-Ti steel ranges from 1270 °C to 1240 °C;
- Low values of the ductility recovery temperature and loss of ductility indicate that the plastic working of 27Mn-4Si-2Al-Nb steel ingots should start at a temperature of approx. 1150 °C;
- 24Mn-3Si-1.5Al-Nb-Ti steel shows higher values of NST, NDT, and DRT. This indicates that the starting temperature of the forming of ingots made of 24Mn-3Si-1.5Al-Nb steel may even be slightly above 1200 °C;
- The developed chemical composition of 27Mn-4Si-2Al-Nb and 24Mn-3Si-1.5Al-Nb-Ti steels, as well as the determined characteristics of the plastic deformation processes at high temperatures, may be the basis for the development of heat-plastic treatment conditions for sheets with high hot ductility and predicted mechanical properties.

## Figures and Tables

**Table 1:** Chemical compositions of investigated steels.

27Mn-4Si-2Al-Nb									
C	Mn	P	S	Si	Al	Nb	Ti	N	O
0.04	27.5	0.002	0.017	4.2	2.0	0.033	–	0.0028	0.0007
**24Mn-3Si-1.5Al-Nb-Ti**									
C	Mn	P	S	Si	Al	Nb	Ti	N	O
0.054	24.4	0.004	0.016	3.5	1.6	0.029	0.075	0.0039	0.0006

## Data Availability

Not applicable.
